# Combining brain stimulation and video game to promote long-term transfer of learning and cognitive enhancement

**DOI:** 10.1038/srep22003

**Published:** 2016-02-23

**Authors:** Chung Yen Looi, Mihaela Duta, Anna-Katharine Brem, Stefan Huber, Hans-Christoph Nuerk, Roi Cohen Kadosh

**Affiliations:** 1 Department of Experimental Psychology, University of Oxford, Oxford, OX1 3UD, United Kingdom; 2 Berenson-Allen Center for Noninvasive Brain Stimulation, Division of Cognitive Neurology, Department of Neurology, Beth Israel Deaconess Medical Center, Harvard Medical School, Boston 02215, MA, USA; 3 Knowledge Media Research Centre, Tübingen, Schleichstrasse 6, 72076, Tuebingen, Germany; 4 Institute of Psychology, Eberhard Karls University, Schleichstrasse 4, 72072, Tuebingen, Germany

## Abstract

Cognitive training offers the potential for individualised learning, prevention of cognitive decline, and rehabilitation. However, key research challenges include ecological validity (training design), transfer of learning and long-term effects. Given that cognitive training and neuromodulation affect neuroplasticity, their combination could promote greater, synergistic effects. We investigated whether combining transcranial direct current stimulation (tDCS) with cognitive training could further enhance cognitive performance compared to training alone, and promote transfer within a short period of time. Healthy adults received real or sham tDCS over their dorsolateral prefrontal cortices during two 30-minute mathematics training sessions involving body movements. To examine the role of training, an active control group received tDCS during a non-mathematical task. Those who received real tDCS performed significantly better in the game than the sham group, and showed transfer effects to working memory, a related but non-numerical cognitive domain. This transfer effect was absent in active and sham control groups. Furthermore, training gains were more pronounced amongst those with lower baseline cognitive abilities, suggesting the potential for reducing cognitive inequalities. All effects associated with real tDCS remained 2 months post-training. Our study demonstrates the potential benefit of this approach for long-term enhancement of human learning and cognition.

Cognitive training offers the potential for individualised learning, prevention of cognitive decline, and rehabilitation^1^. Key aspects that could maximize its potential application beyond the lab include its ecological validity (training design), transfer, and long-term effects. Given that current evidence is mixed, there is an ongoing debate about the transfer and longevity of learning gains from training^1^,^2^. Typical studies involve weeks of repeated training on simple tasks, resulting in restricted ecological validity, i.e., reduced likeliness of repeated training on the same simple task (e.g., non-adaptive, without feedback) as an approach to learning in real-life. Meanwhile, non-invasive brain stimulation (NIBS) has been shown to improve training performance within 5–6 days^3^,^4^, with transfer effects to domains similar to those engaged during training. However, there have been mixed findings on its long-term effects. For example, while some found long-term effects for both trained and non-trained domains^4^, others observed long-term effects only for trained^3^. Given that both cognitive training and neuromodulation could affect neuroplasticity, we hypothesized that their combination would promote greater transfer and long-term effects.

In addition, individual differences have been shown to modulate the effects of cognitive training and NIBS. Although one aim of learning is to reduce cognitive inequalities, cognitive training alone might benefit those with higher cognitive abilities^5^,^6^. Promisingly, NIBS tends to mostly benefit those with lower baseline abilities^7^,^8^,^9^, although further research is needed to examine the influence of ceiling effects^8^,^9^ and possible impairment amongst those with higher baseline abilities^7^. However, current studies have adopted the extreme groups approach whereby participants were classified into groups (e.g., high vs. low) based on a specific cognitive ability, which could affect the reliability and generalisability of the results^10^ (but see^11^). Currently, it is unclear how the parametric effect of individual differences would affect the outcome of a combined approach of cognitive training and NIBS.

In the current study, we combined transcranial direct current stimulation (tDCS), a form of NIBS, and cognitive training (a mathematical video game) to investigate whether their synergistic effects on transfer and long-term change would be greater than training alone. Given our training context and the link between mathematics and working memory^12^, we expected to see transfer effects to this domain. We also examined whether cognitive benefit from this combined approach is influenced by individual baseline cognitive performance. We explored the effects of an hour of combined tDCS and cognitive training, as a previous study showed structural changes after 2 hours of training^13^. We wondered if a shorter training compared to previous studies^3^,^4^ would still show long-lasting effects.

Twenty healthy adults were randomised to receive either 1 mA real or sham tDCS over their bilateral dlPFCs (left: cathodal; right: anodal) during 2 days of 30-minute cognitive training (Fig. 1). Participants trained on an adaptive game featuring fractions and number line estimation, real-life mathematical concepts that are strong predictors of mathematical achievement^14^,^15^. Participants mapped fractions on a virtual number line by moving their body from side-to-side (Fig. 1). Such integration of embodiment within numerical training has been associated with more pronounced training benefits^16^. Moreover, compared to previous studies, the relatively more engaging design (i.e., immediate feedback, adaptive to player performance, training on a more complex task featuring fractions) could increase the attractiveness and likelihood of real-world adoption for learning.

Participants’ performance on the video game was measured by their response times (RT) and accuracy. Note that the duration of our game per day was 30 minutes to coincide with the length of stimulation (30 minutes).

To assess the training effects of our video game independent of tDCS, an active control group was further recruited; they received identical tDCS to the real group while performing non-mathematical visuospatial tasks. To assess long-term effects, a follow-up session was conducted 2 months later, where participants performed their previously assigned tasks (mathematics video game or visuospatial tasks) without tDCS. To examine training effects, transfer effects (working memory) and longevity of all effects, we assessed participants before, immediately after, and 2 months after training.

We examined whether: 1) the real tDCS group would perform better in the video game than the sham tDCS group, with long-lasting improvements; 2) there would be a transfer effect to working memory only in the real tDCS group who trained on the mathematical game; and 3) the effect(s) of tDCS would differ depending on baseline mathematical achievement.

## Methods

### Participants

Thirty healthy right-handed participants (20 females, mean = 24.2 ± SD = 2.1 years) with no history of neurological or psychiatric disorders gave their informed consent to participate in this study. They were assigned to three groups: 1) real tDCS during mathematics video game training (n = 10, 4 males; mean age = 24.6 ± SD = 3.8 years); 2) sham tDCS during mathematics video game training (n = 10, 2 males; mean age = 23.9 ± SD = 2.5 years); and 3) active control group that received real tDCS during non-mathematical visuospatial tasks (n = 10, 4 males; mean age = 24.1 ± SD = 2.0 years). The groups did not differ in terms of gender χ^2^(2) = 1.2, *p* = 0.55. Participants were matched across groups on their performance on a standardized mathematics test (Wechsler Individual Achievement Test, Second UK Edition, WIAT-II UK). Unfortunately, we did not assess participants’ previous experience of video gaming at the individual level to examine if this might have incidentally led to a difference between the groups. This study was given ethical approval by the Berkshire Research Committee and the methods were carried out in accordance with approved guidelines.

### Video game

**Figure 1:**
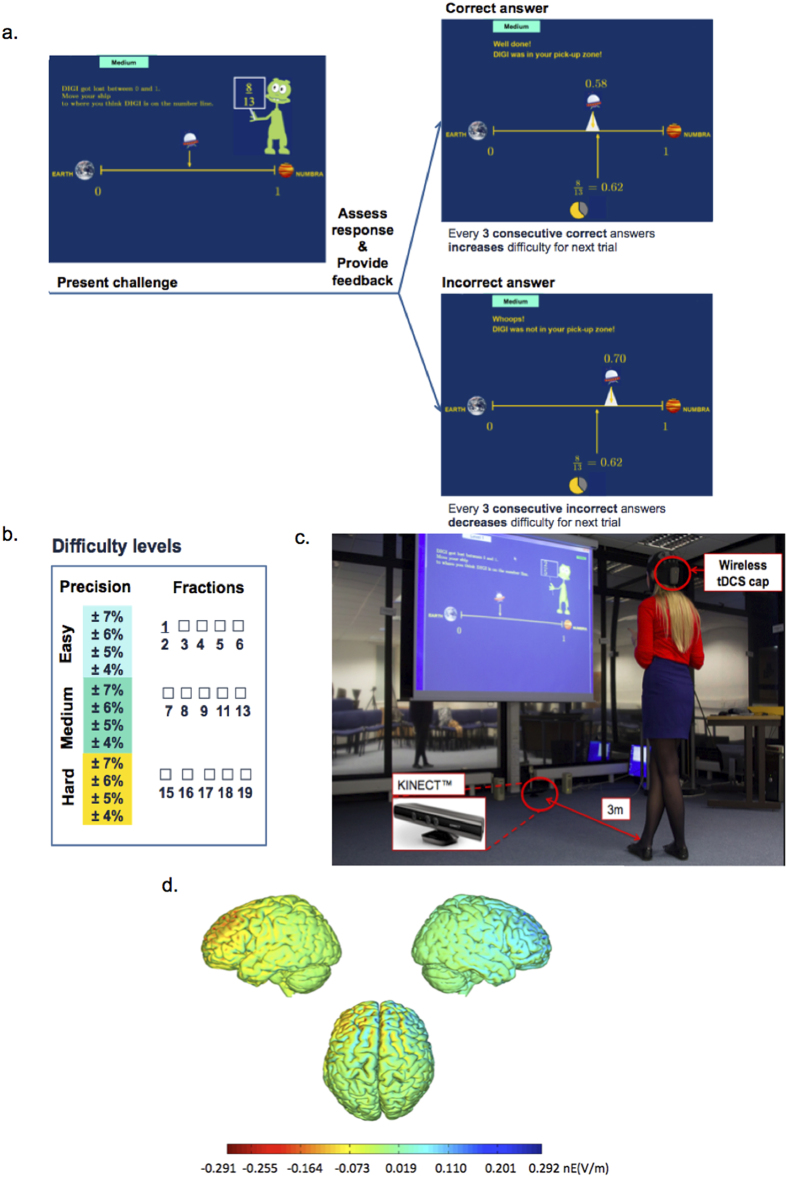
Coupling brain stimulation with a video game to enhance learning: (**a**) Sequence of a trial, adaptive game design. Feedback includes both concrete and abstract numerical representations to strengthen fractions understanding^57^ (For a magnified view, please see Supplementary Figure S3). (**b**) Types of fractions attempted. Depending on participants’ performance, the difficulty was systematically adjusted as a function of fraction category (Easy, Medium, Hard), and precision (accepted deviations from the correct target; ±7%, ±6%, ±5%, ±4% of the number line range). This is to challenge participants to map fractions with greater accuracy, at their maximal capacity. (**c**) Experimental setup included a video game that requires body movements, detected by a motion detector coupled with wireless tDCS. Participants move their bodies side-to-side to locate a spaceship on a virtual number line according to fractions presented on a 1.5 × 1.2 meters screen, 3 meters away from their standing position. (**d**) Computational simulation of current field intensity map over the right-anodal (blue) and left-cathodal (red) dlPFC produced by Neuroelectrics using the modeling engine of the NIC software. Applied currents are maximally concentrated on the cortical surface directly underneath the stimulating electrodes^58^,^59^,^60^.

Based on our previous research that showed more effective training outcomes for training that involves body movements^16^, we designed an adaptive video game that requires participants to indicate the position of fractions on a visually presented number line by moving their body side-to-side. Participants’ movements were captured by a motion-detecting device, KINECT^TM^ (Fig. 1c). Participants performed four practice trials before their first training session. RTs and accuracy (difference between correct and estimated positions on number line) of responses were recorded.

Analyses were performed on data up to level 3 because four participants did not perform beyond this level on the first day (2 tDCS; 2 sham). These levels were categorized based on their level of difficulty: Easy, Medium, and Hard and were selected through a pilot study prior to the current experiment (Fig. 1b) (For full stimulus list of our game, see Supplementary Table S1). Each fraction level was allocated 4 levels of ‘precision’, which specify the amount of deviation from the target allowed for a correct response. The lowest precision corresponds to ±7% allowed deviation based on the number line range, with two intermediate levels defined in steps of ±1%. We chose these specific levels of precision as pilot data indicated that they are appropriate for our targeted population; the easiest level (7%) was the least demanding but it was not too easy, while the most difficult level (4%) was challenging but not unattainable. The levels in between (5%, 6%) provided gradually more challenging trials to stretch the capacity of our participants. Taken together, the level of difficulty was defined by the fraction category and level of precision within that category. The fraction category was defined by the size of the fractions (Fig. 1b), which increase gradually from Easy to Hard and therefore require more precise mapping on the number line. The more difficult fractions do not share the lowest common multiple with the easier fractions (see Supplementary Table S1 for full stimulus list).

The precision requirement was calculated based on the deviation from the target within each category of fractions (Easy, Medium, Hard). In the easiest precision level (7%), a correct answer is accepted as long as it falls within ±7% of the target fraction. In the most demanding precision level (4%), participants were required to map within ±4% of the target to have their trial considered correct. For example, in the Easy level, the fraction 3/5 (0.6) is presented. A response that is considered correct within the 7% level would be 0.558–0.642, and 0.576–0.624 for 4% level. Each trial consisted of a presentation of a fraction challenge, a response window, and an immediate assessment of a response with feedback (Fig. 1a). Participants started the game by mapping positions of Easy fractions with the lowest precision, ±7% from target. After 3 consecutive correct answers, they were promoted to the next level requiring a higher precision of response, i.e., ±6%, followed by ±5% and ±4%. After 3 consecutive incorrect answers, participants were demoted by a category or precision level. For example, participants would be demoted to a lower precision level of 7% if they produced 3 incorrect responses at 6% precision, or to Easy fractions at precision level of 4% if they produced 3 incorrect responses at the Medium level at the precision level of 7%. This game was adaptive in order to challenge participants close to their maximal capacity. Note that to account for all levels of the game achieved by our participants, we conducted an analysis of inter-individual variability in performance based the overall levels achieved by our participants (not restricted to level 3).

### Transcranial direct current stimulation

During real stimulation, 1 mA tDCS was delivered to the bilateral dlPFC (right, F4: anode, left, F3: cathode) for 30 minutes on 2 separate days (within 3 days) during mathematics video game training. The dlPFCs were chosen as stimulation sites, as these are key areas involved in learning^17^ including mathematical learning^4^,^18^ and are hubs for a range of domain-general, executive functions^17^. Therefore, we inferred that these would be ideal stimulation sites to maximise the potential of transfer effects.

We chose to apply a right-anodal, left-cathodal montage for several reasons: 1. Anodal tDCS to the left dlPFC with a reference electrode on the contralateral supraorbital region improved performance on digit span, but only in the forward order^19^. Instead, when repetitive transcranial magnetic stimulation (rTMS) at 1 Hz was applied over the right dlPFC to transiently disrupt its function, performance on both forward and backward digit span were impaired^20^ (note that in contrast to rTMS at 1 Hz, which has an interference effect, the anodal electrode in our study, which is assumed to influence cortical excitability was placed above the right dlPFC); 2. We chose to adopt a bilateral instead of unilateral tDCS montage (stimulating the contralateral dlPFC instead of using a reference electrode) because other studies have shown stronger and more specific effects of the anodal tDCS in bilateral compared to unilateral tDCS^21^,^22^,^23^, and a previous study showed that such montage is more effective in producing localised current flows^24^; and 3. We chose the current montage rather than the opposite montage (right-cathodal, left-anodal) as the latter was shown to impair learning in another training paradigm^25^. Stimulation was delivered via a wireless tDCS cap with two 25 cm^2^ circular sponge electrodes (Neuroelectrics, Barcelona). The current densities were not localised to one hemisphere (Fig. 1d).

The sham group received identical training as the tDCS group, but stimulation was only applied for 30 seconds (15 seconds ramp-up, 15 seconds ramp-down at the beginning and at the end of the training). This brief stimulation is assumed to produce negligible effects on neuronal populations beneath stimulation electrodes, but induces scalp sensations that are indistinguishable from real tDCS^26^,^27^. The active control group was stimulated with the same protocol as the real tDCS group, but during non-mathematical visuospatial tasks (from the Wechsler Abbreviated Scale of Intelligence, WASI-II test).

### Active Control Training

Participants in the active control group were given 15 minutes to perform in each Block Design and Matrix Reasoning subtest of WASI-II (total duration of up to 30 minutes).

In the Block Design task, participants were asked to arrange a few blocks into 10 specified designs or items shown in the Stimulus Book. Each side of the blocks could be purely red, purely white or divided into 2 equal triangles (half red, half white). Participants were given 4 blocks for the first 5 items, and 9 blocks for the following 4 items. They had two practice trials using items 1 and 2 to ensure that they understand the task. They were then required to solve the first 5 items within 60 seconds for each item, and the following 4 items within 120 seconds for each item. They were allowed two trials for the first 2 items, and were given a score of 2 for a correct response on the first trial, a score of 1 for a correct response on the second trial, and a score of 0 for incorrect response on both trials. For the remaining items, they were scored based on the time taken to solve each trial. For items 3–9, they were given a score of 4 for correctly assembled designs within 21–60 seconds, a score of 5 within 16–20 seconds, a score of 6 within 11–15 seconds and a score of 7 within 1–10 seconds. For item 10, they were given a score of 4 for recreating the designs correctly within 66–120 seconds, a score of 5 within 46–65 seconds, a score of 6 within 31–45 seconds and a score of 7 within 1–30 seconds. For each items 11–13, they were given a score of 4 for recreating the designs correctly within 76–120 seconds, a score of 5 within 56–75 seconds, a score of 6 within 41–55 seconds and a score of 7 within 1–40 seconds. The maximum raw score is 71. In the event that the participant had completed his/her blocks in less than 15 minutes, they were required to wear the tDCS cap for the remaining duration while they prepared for the second task, Matrix Reasoning (e.g., filling in their personal details).

In the Matrix Reasoning task, participants were given 30 incomplete matrices or series from the Stimulus Book and were required to select the response option that completes each matrix or series. A correct response was given a score of 1, and an incorrect response was given a score of 0. The maximum raw score is 35. In the event that the participant completed the task in less than 15 minutes, they were required to wear the tDCS cap for the remaining duration.

Please note that, in line with the WASI-II administration guidelines, the active control group did not receive any feedback on their performance and participants’ training was characterized by repeating the task on the next day. The two tasks were not presented in alternated order across or within participants over the three testing days. Overall, each participant completed 10 Blocks Design items and 30 Matrix Reasoning items on each day.

### Cognitive assessments

Cognitive assessments were conducted before, immediately after, and 2 months after training in order to assess the generalizability of training (transfer) and longevity of effects. Participants were tested on their mathematical achievement and working memory capacity (verbal and visuospatial). Mathematical achievement was tested using the Wechsler Individual Achievement Test 2^nd^ UK Edition (WIAT-II, UK). This included tests on numerical operations and mathematical reasoning. The composite scores were used to control for any differences in mathematical achievement during group allocation and to assess the effect of individual differences on training outcomes for real and sham tDCS. Verbal and visuospatial working memory capacities were assessed using Digit Span and Corsi blocks respectively (both forward and backward for each test).

### Statistical Analyses

***Response times and accuracy*** (absolute deviation of answer from target) up to level 3 and within ±3 standard deviations (SD) of the mean were analysed by 4-way mixed analyses of variance (ANOVAs). Time (day 1, day 2) x category (Easy, Medium, Hard fraction problems) x precision (±7%, ±6%, ±5%, ±4% from the exact answer based on the number line range) were the within-subject factors, and group (tDCS, sham) was the between-subject factor. Two months later, data were analysed using a 3-way ANCOVA with category, precision and group. We included day 1 RTs as a covariate to control for baseline performance as it correlated with performance 2 months later (n = 20, Pearson r = 0.72, *p* < 0.01; Spearman r = 0.84, *p* < 0.01). Note that on Day 1, one participant from the tDCS group did not perform up to Level 3, and was therefore excluded from the RT and accuracy analyses and data provided in Table 1.

***Overall performance*** on the game was taken into account when we analysed the relationship between the overall levels (sum of all precision levels in all categories achieved) and participants’ baseline mathematics abilities using both Pearson and Spearman correlation analyses. Note that one participant was excluded for being an outlier (>6 SD from the mean) and when we assessed the gain from the training at the end of the game, performance on day 1 was controlled for as baseline performance, as it was correlated with performance on day 2. We controlled for day 1 instead of subtracting it as it is recommended as the best method suited to our design^28^,^29^.

## Results

We began this experiment with 2 groups, tDCS and sham. To further assess the role of our video game, we conducted an additional experiment (active control group). We report the results from this control experiment at the end of the results section, as participants were not randomly assigned to this group unlike the real and sham tDCS groups. Participants’ game performance was expressed as response time (RT) and accuracy. Working memory was assessed using Digit Span and Corsi Blocks.

### Short-term effects

#### Response times

**Figure 2:**
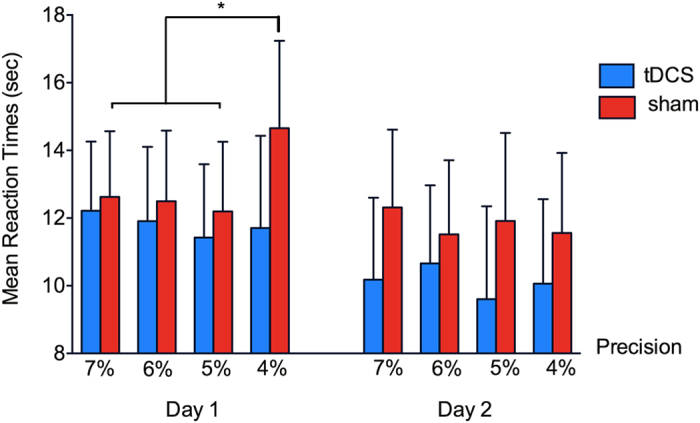
A 3-way interaction between Time, Precision and group for Mean RT of maths training on Day 1 and 2. The source of this interaction was a significant 2-way interaction between Precision and group only for Day 1 (left panel). This interaction was due to a significant difference within the sham group between the hardest precision (4%), and 7% and 5%. Error bars indicate one standard error of mean (SEM).

ANOVA revealed that the more difficult the fractions participants had to map, the longer the RT [F(2,34) = 32.23, *p* < 0.001, η^2^_p_ = 0.66]. There was an interaction between time, precision of fractions mapping, and group [F(3,51) = 4.02, *p* < 0.03, η^2^_p_ = 0.45]. Further analysis revealed that this interaction was due to a significant simple interaction between precision and group on the first day of training [F(3, 51) = 5.23, *p* = 0.003, η^2^_p_ = 0.24]. Post-hoc independent t-tests showed that the precision of the game affected the RT of the sham group on the first day; there was a higher RT in the most difficult precision (4%) compared to other precision levels (5%, 6%, 7%; all *p* < 0.04). In contrast, precision requirements did not affect RT in the real tDCS group [F(3,27) = 1.66, *p* = 0.2, η^2^_p_ = 0.16; difference in the mean RT between the two groups: *p* > 0.2]. The fact that those in the sham group took longer to map fractions within 4% deviation from targets suggests that participants found it difficult to map at this level of precision on day 1. Note that on average, participants first attempted 4% precision (in this case, at Level 1) after 9.8 minutes (SD = 3.3) and performance at precision 4% was averaged across the levels of training (up to Level 3). Therefore, the effect for the most difficult condition occurred at a time point where tDCS is expected to affect neural functions^26^,^30^ and cognitive performance^31^. TDCS did not affect RTs on day 2 (*p* > 0.4) (See Fig. 2).

#### Accuracy

The main effect of time indicated more accurate performance on day 2 [F(1,17) = 12.54, *p* < 0.003, η^2^_p_ = 0.43]. This was dependent on the precision required [time x precision interaction: F(3,51) = 5.48, *p* < 0.002, η^2^_p_ = 0.24]. Notably, there was an interaction between precision and group [F(3,51) = 3.77, *p* < 0.02, η^2^_p_ = 0.18]. While there were no significant differences between tDCS and sham for each precision level (*p* > 0.19), the simple main effect of precision for the sham group was significant [F(1,9) = 17, p = 0.003, η^2^_p_ = 0.65]. Linear trend analysis revealed that 70% of the variance in this interaction was attributed to decreased deviation from the correct location in the sham group with increasing precision demands (linear trend analysis: *p* < 0.001) (Supplementary Figure S1). In contrast, precision demands had no effect on the accuracy of the tDCS group [F(1,8) = 0.001, *p* > 0.98, η^2^_p_ = 0.001, linear trend analysis: *p* > 0.58] (See Supplementary Figure 1). Noticeably, the tDCS group showed a trend toward shorter RTs at the most demanding precision level (4%), but with lower accuracy. Further analysis indicated that this was not due to a speed-accuracy trade-off (r = 0.02, *p* > 0.91).

### Number of trials

Participants attempted the following number of trials on Day 1: 102 (SD = 26), Day 2: 101 (SD = 28) and 2 months later: 104 (SD = 23). A 4-way ANOVA with day (1,2) x level (3) x precision (4) x condition (real, sham) was conducted on the number of trials up to level 3, as 4 participants did not perform beyond level 3. All interactions with condition were not significant, all *p* > 0.8. The main effect of day and precision was significant [F(1,17) = 6.49, p = 0.021, n^2^_p_ = 0.28 and F(3,51) = 13, p < 001, n^2^_p_ = 0.43 respectively].

#### The influence of individual differences

**Figure 3:**
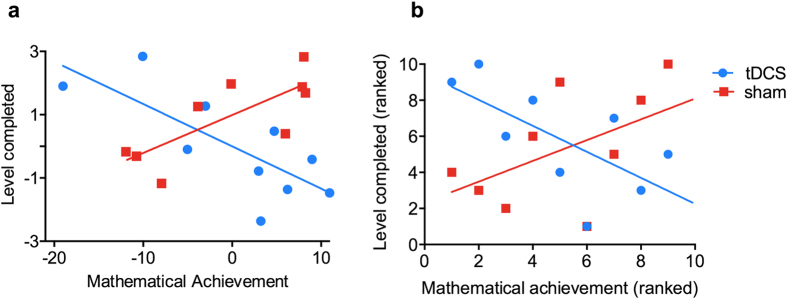
Double dissociation in stimulation and cognitive training outcomes: combined tDCS and cognitive training is more beneficial for low achievers, while cognitive training alone is more beneficial for high achievers. (**a**) Pearson and (**b**) Spearman correlational analyses showing the modulatory effects of baseline mathematics achievement and levels completed at the end of the training. The data on panel A reflects residuals after partialling out the correlation with the performance on the first day of training. One participant from the sham group was excluded for underperforming by 2.5 SD from the mean. Note that some data points on B panel are overlapping and therefore less data points appear.

As predicted, individual baseline ability modulated the effects of training (Fig. 3a). In the sham group, those with higher mathematics achievement achieved more levels during training (r = 0.77, *p* < 0.01). This is consistent with the typical observation that ‘those who start ahead, stay ahead’^5^, or the so-called “Matthew effect”^32^, and with previous studies investigating cognitive training^6^,^16^,^33^. Notably, the tDCS group showed an opposite pattern; those with lower baseline mathematics achievement progressed through more levels during training (r = −0.76, *p* < 0.01). These correlations differed between groups (Steiger’s Z-test, Z = 3.62, *p* = 0.002)^34^. Similar results were achieved with Spearman correlations [sham: r = 0.57, *p* < 0.08; tDCS: r = −0.72, *p* < 0.02] (Fig. 3b). These observations cannot be explained by ‘ceiling effects’ as both sham and tDCS groups achieved on average similar levels at the end of training (*p* > 0.32). An explanation based on regression to the mean is also unsatisfactory, as it cannot explain the differences in the slopes between both groups^35^.

#### Working memory

Working memory (WM) performance pre- and post-training were analysed by a 4-way ANOVA with task (verbal WM, visuospatial WM) x order of retrieval (forward, backward) x time (pre-training, post-training) as within-subject factors, with group (tDCS, sham) as a between-subject factor.

There was an interaction between the type of working memory (WM) task (verbal, visuospatial), time, and group [F(1,18) = 22.4, *p* < 0.0001, η^2^_p_ = 0.56]. Note that order of retrieval (forward vs. backward) did not contribute to this 3-way interaction (*p* > 0.38). Therefore, it is suggested that, in these cases, the analysis should not decompose the interaction into backward and forward to avoid type I error. Nevertheless, for visual illustration, we present the interaction of time x group for verbal WM separately for backward and forward spans in Supplementary Figure S2, which shows a similar pattern.

The 3-way interaction between task, time and group was decomposed for verbal and visuospatial WM.

#### Verbal WM

**Figure 4:**
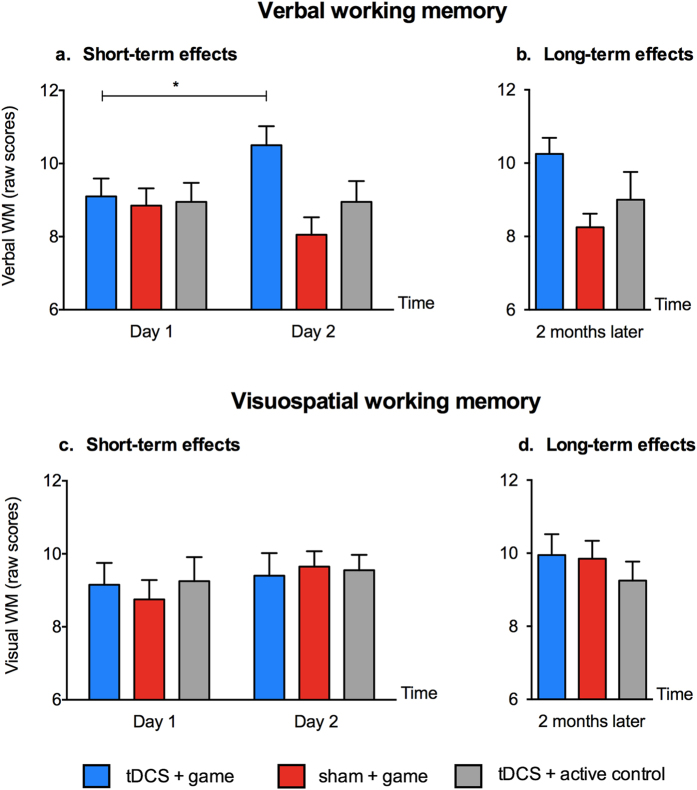
Transfer of gains from video gaming coupled with tDCS. (**a**) Only those who received real tDCS showed increased verbal WM capacity immediately after training, and (**b**) 2 months later. (**c**) There were no improvements in the visuospatial WM capacity of participants either immediately after training or d) 2 months later. Data are represented as mean ± SEM.

There was an interaction between time and group [F(1,18) = 10.62, *p* < 0.004, η^2^_p_ = 0.37] (Fig. 4a). TDCS and sham groups did not differ in capacity at pre-test [t(18) = 0.37, *p* > 0.72], but at post-test, the tDCS group showed an improved verbal WM capacity compared to the sham group [t(18) = 3.46, *p* > 0.002, Cohen’s d = 0.63]. Post-hoc analyses revealed that only the tDCS group improved in verbal WM capacity [tDCS: t(9) = 3.09, *p* < 0.01, Cohen’s d = 0.72], while sham group performance did not change (note that the decline observed in the sham group was not significant: t(9) = 0.8, *p* > 0.44).

#### Visuospatial WM

The simple main effects, as well as the interaction between time and group were not significant [*F* < 2.42, *p* > 0.14] (Fig. 4c).

### Long-term effects (2 months post-training)

#### Response times

At follow-up, the tDCS group was 2.4 seconds (20%) faster than the sham group in the game [group: F(1,17) = 4.84, *p* < 0.04, η^2^_p_ = 0.22] (Table 1). This was based on a 3-way Analysis of Covariance (ANCOVA) with level, precision and group on RT two months post-training.

There was a significant linear trend in the RT observed across time (day 1, day 2, 2 months later), [F(1,18) = 10.29, *p* < 0.005, n^2^_p_ = 0.36], although the interaction with group showed only a trend, [F(1,18) = 3.12, *p* < 0.094 n^2^_p_ = 0.15]. Further analyses on separate groups indicated that there was a significant linear trend in the tDCS group, [F(1,9) = 8.74, *p* < 0.032, n^2^_p_ = 0.49, corrected for multiple comparisons, due to the non-significant interaction] but not in the sham group [F(1,18) = 1.78, *p* > 0.43 n^2^_p_ = 0.17].

#### Accuracy

A significant interaction between precision and group [F(3,48) = 6.28, *p* < 0.001, η^2^_p_ = 0.28, controlling for accuracy on day 1 was due to more precise mapping fractions in the tDCS group compared to the sham group in the easiest precision level [t(18) = 2.71, *p* < 0.01, Cohen’s d = 0.54] (For accuracy values, see Table 1).

#### Number of trials

When the follow-up data was included, the interaction between precision and condition was significant, F(3,51) = 17.21, *p* = 0.035, n^2^_p_ = 0.15. However, when decomposed, we did not find any significant differences between the groups at any precision level, all *p* > 0.15.

#### Working memory

The tDCS group showed a sustained effect in verbal WM capacity after two months [t(18) = 3.45, *p* < 0.002, Cohen’s d = 0.63] (Fig. 4b), but no effect in visuospatial WM capacity [t(18) = 0.13, *p* = 0.89] (Fig. 4d). Results replicated those immediately after training, indicating that the type of WM enhancement depended on the group [F(1,18) = 6.13, *p* < 0.02, η^2^_p_ = 0.44] (For WM scores, see Supplementary Table S2).

### The role of training in tDCS effect

In order to assess whether the coupling of tDCS and the mathematical game was critical to these effects on working memory and not a result of stimulating the dlPFC *per se*, we conducted a separate experiment. We recruited an active control group (n = 10; matched across age and baseline mathematics achievement) who trained on Matrix Reasoning and Block Design (See Methods) while receiving real tDCS. Both Matrix Reasoning and Blocks Design tasks engage visuospatial skills but non-mathematical skills. However, due to the nature of these two tasks, their difficulty levels cannot be directly and accurately compared. We measured performance on both tasks using raw and scale scores (Supplementary Table S3). Performance was timed and the overall time taken was about 30 minutes. These tasks served as ”placebo training” (not the main training, but included visuospatial processing similar to our game). This allowed us to 1) assess the specific training effects of our mathematical video game, and 2) control to some extent, the cognitive processes engaged during the stimulation period^36^,^37^. Note that the primary role of the active control group was to enable the comparison of verbal and visuospatial WM changes with those observed in the real and sham tDCS groups. The active control group did not receive any training on the maths video game and therefore, we did not have any accuracy or RTs for this group (for further analyses, see Supplementary Results). When we added the results from this group to the ANOVA: time (day1, day 2) x group (real tDCS, sham tDCS, active control), the reported interaction between the types of WM task, time and group remained significant [F(1,28) = 15.41, *p* = 0.012, n^2^_p_ = 0.21] (Fig. 4). We therefore decompose the interaction to the different WM tasks.

#### Verbal WM

The interaction between time and group was significant [F(2, 28) = 7.89, *p* = 0.009, n^2^_p_ = 0.22]. The tDCS and control groups (both sham and the active control groups) differed in verbal WM at post-testing [t(28) = 3.07, *p* = 0.005]. Only the tDCS group showed improved WM capacity at post-testing [tDCS: t(9) = −3.1, *p* < 0.01; control groups: t(19) = 1.02, *p* = 0.32].

#### Visuospatial WM

There was no interaction between time and group (*p* > 0.59, n^2^_p_ = 0.01).

## Discussion

In the current study, we examined whether 60 minutes of combined brain stimulation and mathematical video gaming could 1) improve training performance with long-term effects; 2) induce transfer to working memory and 3) modulate training outcomes based on initial mathematical abilities. The key findings and their implications are reviewed below.

As predicted, brain stimulation further improved training performance, with immediate and long-term gains. This improved performance is especially apparent between the two groups with increasing difficulty levels; the sham group showed an expected decline in performance, while those who received tDCS did not. This supports the finding that tDCS is more effective when participants are engaged in a difficult task^38^. Only after two days of 30-minute training, those who received tDCS were on average 9% quicker in mapping fractions, and continued to improve by another 11% when tested 2 months later without further practice and use of tDCS (linear trend analysis: tDCS, *p* < 0.02; sham, *p* > 0.2, see also Results and Table 1). This delayed benefit post-anodal tDCS expands on that reported by Floel and colleagues who found behavioural improvements in memory only after 1 week post-training^39^. The further improvement in performance by 8% at follow up is also consistent with the findings of Reis and colleagues who showed that anodal tDCS during training induced positive offline gains compared to sham^40^. Such gains were thought to be due to enhanced protein synthesis following anodal tDCS during training, or the result of interaction between the excitability effects during and after training, and downstream of learning- related protein synthesis^40^. Indeed, when assessed 2 months later without any brain stimulation, the tDCS group showed about 18% faster responses than their initial performance, while the sham tDCS group showed only 7% improvement (Table 1). Together, these findings suggest that 2 days of 30-minute training with 1 mA tDCS could have long-lasting impact on neuroplasticity. Further studies could collect physiological data to investigate the exact mechanisms of the effects observed in this study.

On the subject of transfer, we found improvements in working memory capacity, a domain not directly trained. Participants in the tDCS group retained at least one item more than those in the control groups immediately after training, and retained this benefit over a period of 2 months without further training and tDCS. This is a novel finding with potential practical implications, given the brevity of training and its transfer impact at the capacity level. Studies that have shown similar improvements took up to 100 hours of training^41^. It is important to point out that this transfer effect is exclusive to the combination of tDCS and our mathematical video game training. No transfer effect was found in the active control group that trained on visuospatial tasks or the sham tDCS group (see Supplementary Results for further analyses). Therefore, our results suggest that the mechanisms behind these observations are not due to tDCS *per se*, but an outcome of the synergy between tDCS and our training game. Notably, the transfer effect to verbal working memory suggests the predominant use of verbal components as a strategy during our game. Indeed, participants had to retain symbolic fractions in mind in order to map them as precisely as possible on the number line. Moreover, fractions problem solving and mathematical computation are linked to verbal working memory^42^,^43^. Another possible explanation is that, as digit span forward and backward engage an overlapping functional neural system linked with working memory, specifically the right dlPFC^44^, anodal tDCS over the right dlPFC during our video game might have reinforced synaptic efficiency between dlPFC and overlapping networks, as suggested in previous brain stimulation and neuroimaging work^40^,^45^,^46^. Further studies could investigate whether this effect is due to enhanced protein synthesis during training, which has been proposed to trigger downstream interactions of learning-related protein in motor learning^46^. As for the lack of improvement of the active control group, it is difficult to explain why a null result was obtained. The inclusion of an active control group in our study was to assess whether the effect we observed on working memory was due to tDCS *per se,* and therefore it included similar cognitive processing, without numerical information. The null results could be attributed to the specific nature of training, stimulation of this montage, or other factors. However, the lack of WM improvement in the active control group does not impact on the potential connection between working memory and fluid intelligence^47^, which is beyond the scope of the current study’s aims and design.

Finally, as predicted, cognitive gains from training and brain stimulation were influenced by individual baseline mathematical achievement. There was an opposite pattern in the correlation between game performance and baseline achievement between the sham and tDCS groups. In the sham group, those with higher baseline achievement progressed through more levels in the game. This pattern mirrors performance in a typical classroom^5^,^32^ and findings of previous cognitive training studies^6^. On the contrary, those with lower baseline achievement achieved more levels in the game in the tDCS group. These findings corroborate and build on previous findings that tDCS efficacy is influenced by baseline performance in a categorical fashion (low vs. high mathematics anxiety^7^; low vs. high visual short-term memory^8^; trained vs. untrained musicians^9^). The observed relationship is in line with the prediction that greater cognitive gains for those with lower baseline abilities will be achieved via modulation of cortical excitation and inhibition using methods like tDCS^48^. One possible explanation is that the effects of tDCS are more salient for difficult trials since that performance on easy trials are likely to be close to optimal response, and tDCS is less likely to produce further, significant behavioural improvements^49^. It might be that, compared to participants with higher achievement, those with lower achievement were more likely to have found our task more difficult, and hence benefited more from tDCS. The lack of effects among individuals with higher achievement might also be capped by individual limits of cortical excitability^7^,^8^ (see also^48^ for a brief review). Given that the physiological effects of tDCS on human cognition are likely to be complex, further research using multi-modal approaches (e.g., neuroimaging and computational simulations) is needed to understand the mechanisms underlying these observations^49^.

Taken together, these findings present two new contributions to current literature. Firstly, when assessing the efficacy of NIBS, inter-subject variation in baseline cognitive achievement should be taken into account. This might be especially relevant for stimulation over areas that are more strongly modulated by genetic factors such as the dlPFC^50^,^51^. By modelling the interactions between NIBS and individual brain characteristics, we could refine our understanding of the mechanisms of NIBS. Secondly, by finding that tDCS could reduce rather than increase existing disparities in performance, our study addresses concerns about the potential risk of NIBS for increasing cognitive inequality in healthy individuals^52^,^53^. Further research should determine how individual differences and tDCS would interact in other cognitive domains and populations.

One limitation of the current study is its relatively modest sample size. This sample size is comparable however, to other cognitive studies using tDCS^54^. In spite of this, we had sufficient power to detect group differences and interactions^55^, and the effect sizes that we found are similar to that reported in the literature on the use of tDCS in cognitive studies^56^. The current findings could motivate future studies to test larger samples and examine the underlying changes at the neural level. Another open question is whether the improvement we observed on the game was due to arithmetic or visuospatial accuracy. Due to the nature of our task, it is difficult to disentangle these two cognitive components, which are interlinked and involved in numerical mapping. Another control task that could tease apart the contribution of each component is needed.

In sum, we show that short- and long-term enhancement of human cognitive functions is achievable within the timescale of an hour. Conceptually, we demonstrated that by combining brain stimulation and training with good ecological validity, we could achieve synergistic effects that are greater than those achieved separately. In practical terms, these findings highlight the possibility of achieving long-term cognitive enhancement with reduced time investment using combined non-pharmacological methods. Identifying inter-individual variations in response to such interventions could contribute towards understanding the mechanisms underlying such interactions, efficacy of such methods on different individuals, and eventually, the design of individualised stimulation. Overall, this study offers new directions for research on the mechanisms of neuroplasticity, learning, and the influence of individual differences on cognitive enhancement.

## Additional Information

**How to cite this article**: Looi, C. Y. *et al.* Combining brain stimulation and video game to promote long-term transfer of learning and cognitive enhancement. *Sci. Rep.* **6**, 22003; doi: 10.1038/srep22003 (2016).

## Supplementary Material

Supplementary Information

## Figures and Tables

**Table 1:** Game RT and accuracy with improvements within- and between groups.

RT (Raw values)			
	Day 1	Day 2	2 months later
tDCS	11.81 (4.27)	10.79 (3.43)	9.63 (2.91)
Sham	12.99 (3.45)	11.82 (4.85)	12.07 (4.02)
**Improvement** ***within*** **groups (%)**			
**Time**	**Day 2 vs. Day 1**	**2 months later vs. Day 2**	**2 months later vs. Day 1**
tDCS	8.6	10.8	18.5
sham	9	−2.1	7
**Difference** ***between*** **groups (tDCS-sham, %)**			
**Time**	**Day 1**	**Day 2**	**2 months later**
	1.7	8.7	20.3
**Accuracy (Raw Values)**			
	**Day 1**	**Day 2**	**2 months later**
tDCS	0.0327 (0.0071)	0.0287 (0.0072)	0.0271 (0.0081)
Sham	0.0343 (0.0093)	0.0287 (0.0054)	0.03000 (0.0059)
**Improvement** ***within*** **groups (%)**			
**Time**	**Day 2 vs. Day 1**	**2 months later vs. Day 2**	**2 months later vs. Day 1**
tDCS	12.2	5.6	17.1
sham	16.3	4.5	12.5
**Difference** ***between*** **groups (tDCS-sham, %)**			
**Time**	**Day 1**	**Day 2**	**2 months later**
	0.5	0	0.1

Improvements within and between groups (tDCS vs. sham) are shown as % changes. Data are depicted as mean ± SD.
