# Supplementary material for: Combining brain stimulation and video game to promote long-term transfer of learning and cognitive enhancement
Source: Sci Rep. 2016 Feb 23;6:22003. doi: 10.1038/srep22003 (PMC4763231; doi:10.1038/srep22003)
Supplement: Supplementary Information [file srep22003-s1.doc]

**SUPPLEMENTARY INFORMATION**

**Combining brain stimulation and video game to promote long-term transfer of learning and cognitive enhancement**

Chung Yen Looi, Mihaela Duta, Anna-Katha­­­rine Brem, Stefan Huber, Hans-Christoph Nuerk, and Roi Cohen Kadosh^*^

**FIGURES**

**Figure S1.** Accuracy as a function of precision and group. Note the linear trend in the decreased deviation in the sham group. Differences between groups at 4% precision were not significant (t(18)=1.35, *p*=.19). Data are depicted as mean ± one SEM.


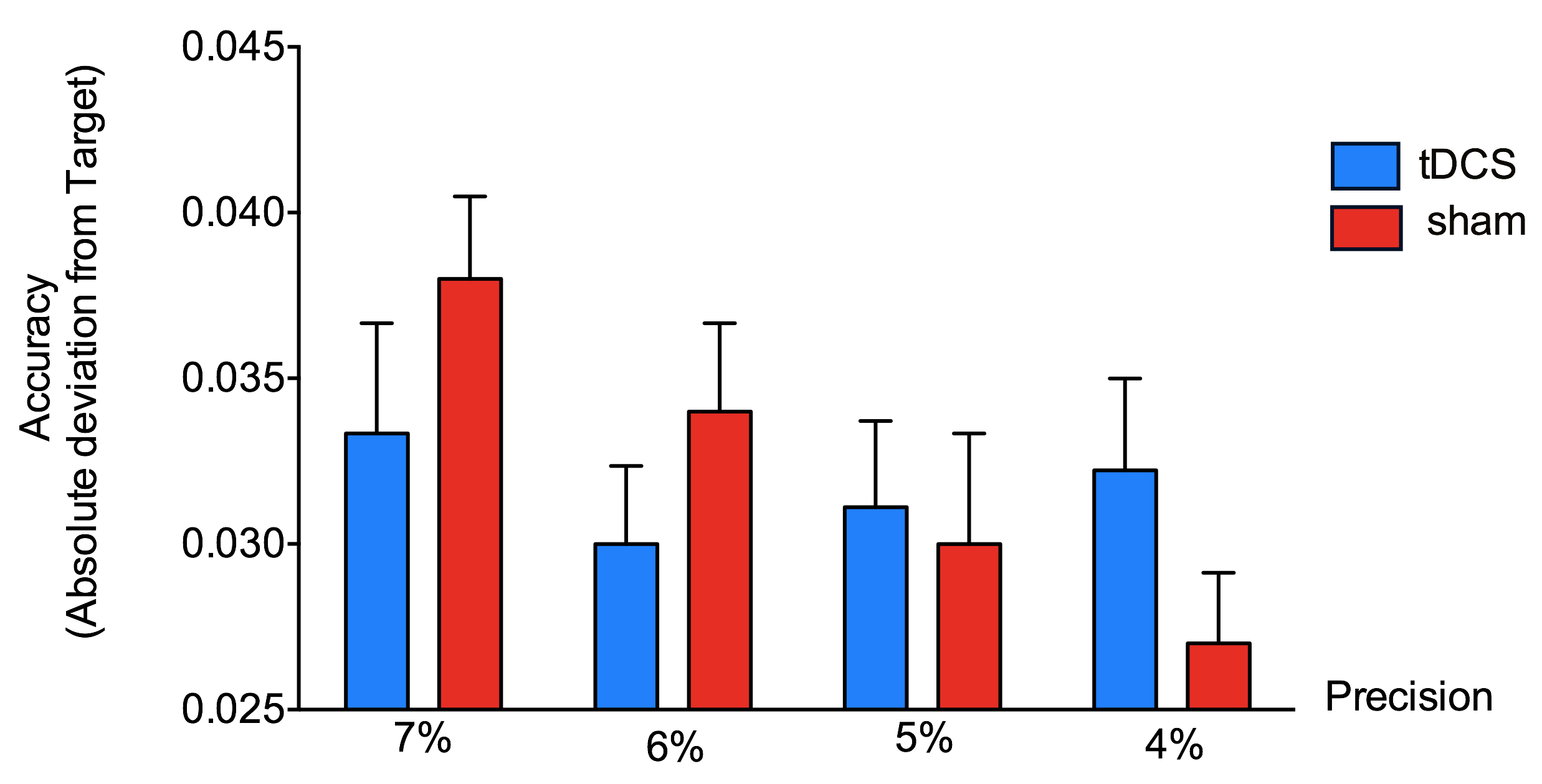


**Figure S2** Verbal WM forward and backward. Data are depicted as mean ± one SEM.


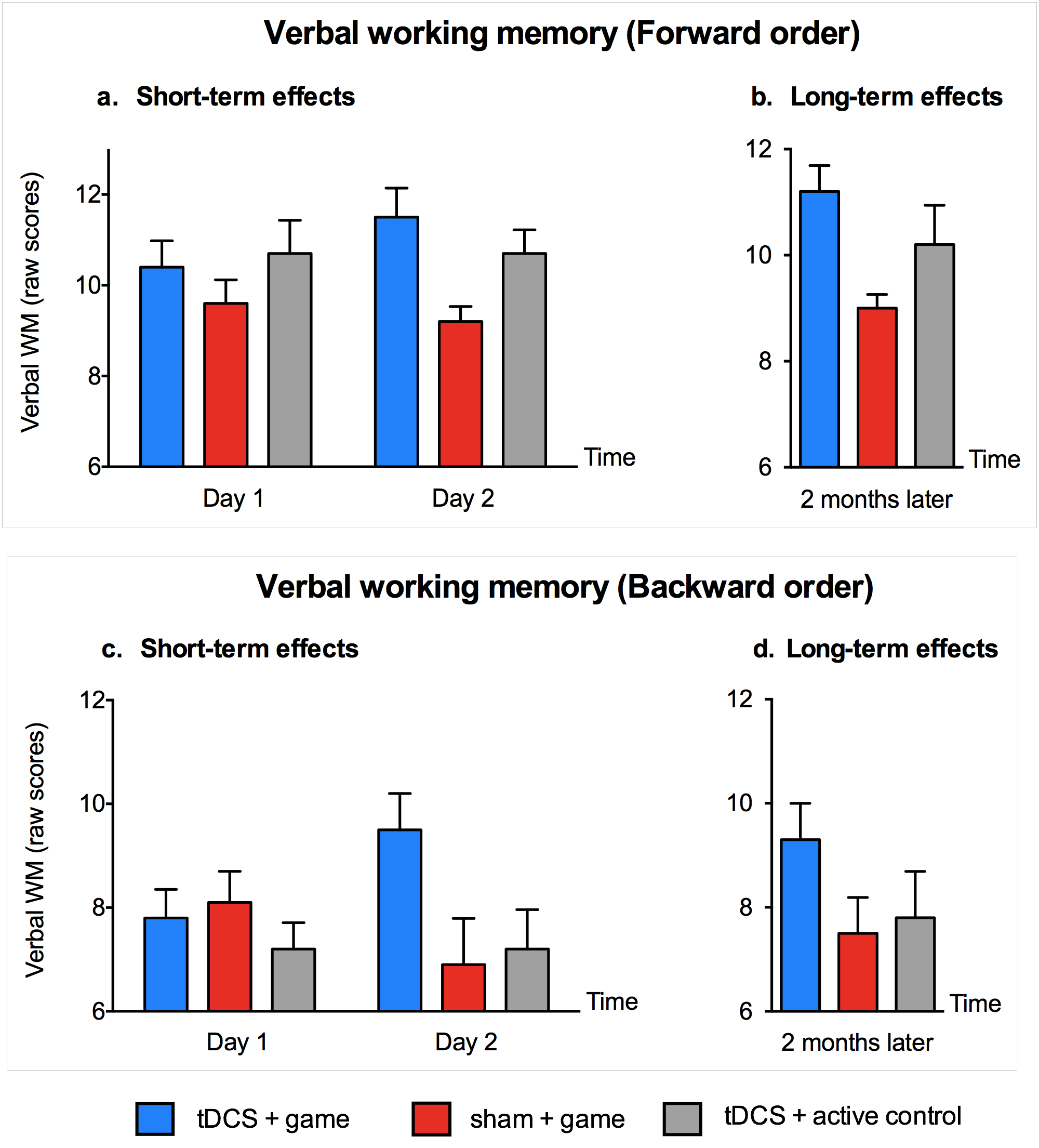


**Figure S3.** An example of a trial (Source of all earth images: NASA).


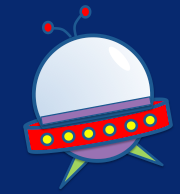

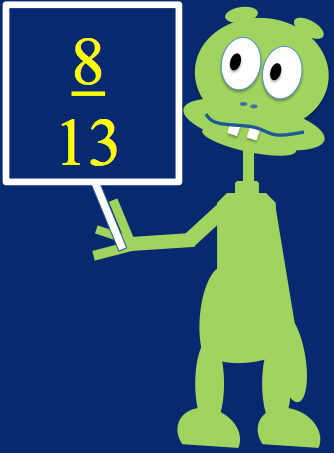

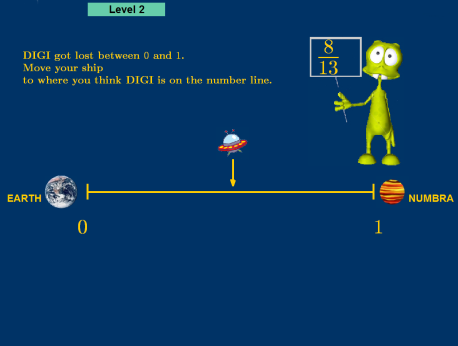
Presentation of challenge:

**Medium**

Example for feedback for a correct answer:


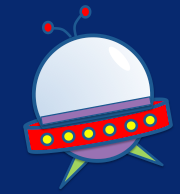

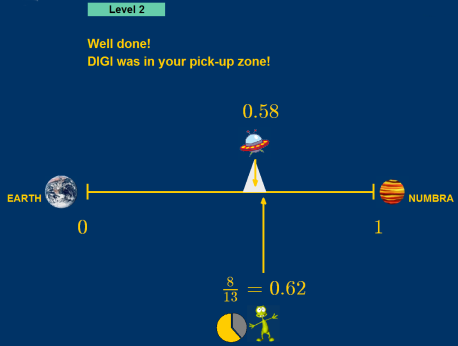


**Medium**

Example for feedback for an incorrect answer:


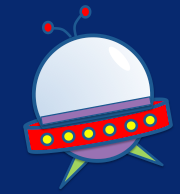

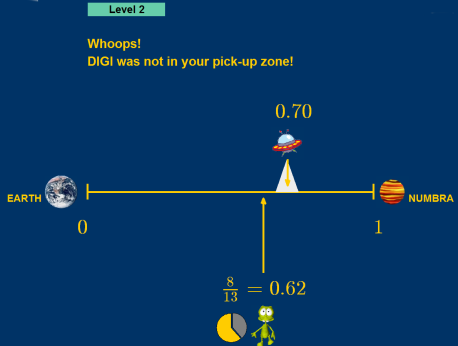


**Medium**

**TABLES**

**Table S1.** Table showing the list of fractions used in the mathematics video game.

| **Level** | **Number line range (anchors)** | **Range of fractions numerators** | **Range of fractions denominators** | **Description** |
| --- | --- | --- | --- | --- |
| 1 | 0-1 | 1-5 | 2-6 | Simple fractions for all possible combinations |
| 2 | 0-1 | 2-9 | 7-11 | Simple fractions for all possible combinations |
| 3 | 0-1 | 2-16 | 15-19 | Simple fractions for all possible combinations |
| 4 | 0-1 | 3-19 | 21-29 | Two simple fractions each for denominators ranging from 21-29. Half of the numerators are even numbers. |
| 5 | 3/32-1 | 5-32 | 31-41 | Two simple fractions each for denominators ranging from 31-41. Half of the numerators are even numbers. |
| 6 | 3/32-1 | 12-34 | 31-41 | Two simple fractions each for denominators ranging from 31-41. Half of the numerators are even numbers. The initial anchor and the target needs to be converted to fractions with common denominators |
| 7 | 5/48-39/42 | 1-19 | 3-45 | All possible sets of fractions where both anchors and targets need to be converted to fractions with common denominators. |

**Table S2.** Raw scores of verbal working memory (Digit span) and visuospatial working memory (Corsi Blocks). Data are depicted as mean ± SD.

| **Task** | **Verbal WM** | | | **Visuospatial** | | |
| --- | --- | --- | --- | --- | --- | --- |
| Time  Group | Pre-training | Post-training | Follow-up (2 months) | Pre-training | Post-training | Follow-up  (2 months) |
| tDCS  + game | 9.1  (1.56) | 10.5 (1.65) | 10.25  (1.4) | 9.15  (1.90) | 9.4  (1.96) | 9.95  (1.8) |
| sham + game | 8.85 (1.49) | 8.05 (1.52) | 8.25  (1.18) | 8.75  (1.67) | 9.65  (1.31) | 9.85  (1.55) |
| tDCS + active control | 8.95 (1.66) | 8.95 (1.82) | 9.0  (2.39) | 9.25  (2.09) | 9.55  (1.32) | 9.25  (1.64) |

**Table S3.** Raw and scaled scores of Blocks Design and Matrix Reasoning. Data are depicted as mean ± SD.

| **Blocks Design** | | | |
| --- | --- | --- | --- |
|  | Pre-training | Post-training | Follow-up (2 months) |
| Raw score | 57.1 (13.2) | 61.6 (10.9) | 62.4 (13.3) |
| Scaled Score | 59.4 (9.0) | 62.2 (7.5) | 63.1 (8.9) |
| **Matrix Reasoning** | | | |
|  | Pre-training | Post-training | Follow-up (2 months) |
| Raw score | 27.5 (4.7) | 29.2 (4.3) | 29.4 (3.6) |
| Scaled Score | 54.3 (9.4) | 57.7 (8.4) | 58.1 (7.1) |

**Supplementary Results**

**Active control group training**

**Short-term effects**

**Blocks Design**

**Raw Scores**

Repeated measures ANOVA with Day (1,2) was conducted and there was a significant improvement from Day 1 (mean=57.10, SD=13.15) to Day 2 (mean=61.60, SD=10.94), F(1,9)=8.19, p<.02, η^2^_p_=.48.

**Standardised scores**

Repeated measures ANOVA with Day (1,2) was conducted and there was a significant improvement from Day 1 (mean=59.40, SD=8.97) to Day 2 (mean=62.2, SD=7.48), F(1,9)=5.9, p<.04, η^2^_p_=.4.

**Matrix Reasoning**

**Raw Scores**

Repeated measures ANOVA with Day (1,2) was conducted and there was a significant improvement from Day 1 (mean=27.50, SD=4.74) to Day 2 (mean=29.2, SD=4.26), F(1,9)=18.45, p<.002, η^2^_p_=67.

**Standardised scores**

Repeated measures ANOVA with Day (1,2) was conducted and there was a significant improvement from Day 1 (mean=54.30, SD=9.42) to Day 2 (mean=57.7, SD=8.39), F(1,9)=18.45, p<.002, η^2^_p_=67.

**Long-term effects within group**

**Blocks Design**

**Raw Scores**

ANOVA was conducted with day (2, 2 months later) and day 1 as covariate because Day 1 and 2 months later was strongly correlated (n=10, Pearson r=.92, p<.001, Spearman r=.93, p<.001).

There were no significant differences in the scores on day 2 and 2 months later, F(1,8)=.87, p=.38 (day 2, mean=61.6 SD=10.94 and 2 months later, mean=62.40, SD=13.31).

**Standardised scores**

ANOVA was conducted with day (2, 2 months later) and day 1 as covariate because Day 1 and 2 months later was strongly correlated (n=10, Pearson= r=.92, p<.001, Spearman r=.89, p=.001).

There were no significant differences in the scores on day 2 and 2 months later, F(1,8)=.78, p=.4 (day 2, mean=62.2 SD=7.48 and 2 months later, mean=63.10, SD=8.9).

**Matrix Reasoning**

**Raw scores**

ANOVA was conducted with day (2, 2 months later) and day 1 as covariate because Day 1 and 2 months later was strongly correlated (n=10, Pearson= r=.92, p<.001, Spearman r=.86, p=.002).

There were no significant differences in the scores on day 2 and 2 months later, F(1,8)=1.44, p=.27 (day 2, mean=29.20 SD=4.26 and 2 months later, mean=29.40, SD=3.6).

**Standardised scores**

ANOVA was conducted with day (2, 2 months later) and day 1 as covariate because Day 1 and 2 months later was strongly correlated (n=10, Pearson= r=.92, p<.001, Spearman r=.76, p=.011).

There were no significant differences in the scores on day 2 and 2 months later, F(1,8)=1.35, p=.28 (day 2, mean=57.70 SD=8.39 and 2 months later, mean=58.10, SD=7.11).
